# The use of radiosensitizing agents in the therapy of glioblastoma multiforme—a comprehensive review

**DOI:** 10.1007/s00066-022-01942-1

**Published:** 2022-05-03

**Authors:** Niklas Benedikt Pepper, Walter Stummer, Hans Theodor Eich

**Affiliations:** 1 Department of Radiation Oncology, University Hospital Muenster, Albert-Schweitzer-Campus 1, Building A1, Muenster, Germany; 2 Department of Neurosurgery, University Hospital Muenster, Albert-Schweitzer-Campus 1, Building A1, Muenster, Germany

**Keywords:** Glioblastoma, Radiation therapy, Radiosensitizer, Review

## Abstract

**Background:**

Glioblastoma is the most common malignant brain tumor in human adults. Despite several improvements in resective as well as adjuvant therapy over the last decades, its overall prognosis remains poor. As a means of improving patient outcome, the possibility of enhancing radiation response by using radiosensitizing agents has been tested in an array of studies.

**Methods:**

A comprehensive review of clinical trials involving radiation therapy in combination with radiosensitizing agents on patients diagnosed with glioblastoma was performed in the National Center for Biotechnology Information’s PubMed database.

**Results:**

A total of 96 papers addressing this matter were published between 1976 and 2021, of which 63 matched the subject of this paper. All papers were reviewed, and their findings discussed in the context of their underlining mechanisms of radiosensitization.

**Conclusion:**

In the history of glioblastoma treatment, several approaches of optimizing radiation-effectiveness using radiosensitizers have been made. Even though several different strategies and agents have been explored, clear evidence of improved patient outcome is still missing. Tissue-selectiveness and penetration of the blood–brain barrier seem to be major roadblocks; nevertheless, modern strategies try to circumvent these obstacles, using novel sensitizers based on preclinical data or alternative ways of delivery.

## Introduction

Glioblastoma multiforme (GBM) is the most common malignant brain tumor in adults. Today’s standard therapy involves resective surgery as well as adjuvant chemoradiation followed by subsequent chemotherapy. But even though research in this field has improved patient outcome by optimizing surgical as well as adjuvant strategies [1–3], overall prognosis of this tumor entity remains poor. Clinical courses are typically branded by local relapse adjacent to the primary tumor site, often limiting the therapeutic possibilities of re-resection or re-administration of radiation therapy (RT).

Several attempts were made to improve patient outcome by enhancement of radiation response via radiosensitizers. With multiple possible approaches of targeting this issue, for instance by reducing radioresistance deriving from tumor hypoxia, generating reactive oxygen species, or interfering with the repair of radiation-induced DNA damage, an array of agents have been evaluated in clinical trials in this regard. Nevertheless, the prognosis of glioblastoma remains inadequate in the 21st century. The paper at hand aims to give a comprehensive overview over past approaches, findings and problems concerning radiosensitizing agents in glioma therapy, as well as the possible future directions in this field.

## Methods

A comprehensive search was performed in the National Center for Biotechnology Information’s (NCBI) PubMed database, via advanced search. Studies were included or excluded based on the presence or absence of a therapy scheme including a radiosensitizing agent during radiation therapy on one or multiple cohorts of patients that included diagnosed glioblastoma multiforme. The source was last consorted 18 December 2021. MeSH (medical subject headings) terms as well as unspecified keywords were combined in the search term ((glioblastoma multiforme[All]) OR (glioblastoma[MeSH Terms])) AND ((radiosensitizer[MeSH Terms]) OR (radiosensitizing[All])) AND ((radiation therapy[MeSH Terms]) OR (ionizing radiation [All])), filtering for all clinical trials on patients with glioblastoma multiforme with involvement of radiosensitizing agents. Trials were grouped together based on the respective agent and underlying mechanism and trial data were summarized, specifying dosing of the radiosensitizer as well as the radiation regimen, patient number and outcome. Not available (N/A) data were labeled as such.

## Results

A total of 96 publications on clinical trials involving radiosensitizing agents and glioblastoma were registered between 1976 and 2021. All publications were reviewed for relevance regarding the paper at hand. 33 publications were excluded because of unfitting context—several of these trials evaluated the issue of photosensitization in glioma therapy. Since this matter is not only related to the subject of this review, but the administered agent (5-aminolevulinic acid) might also play a role in future developments of radiosensitizing research, it will be addressed separately at the end of the discussion. The remaining 63 clinical trials were assessed and will be discussed with reference to the underlying mechanisms concerning their findings as well as subsequent developments in the use of the specific agent in glioblastoma therapy.

The different agents addressed in this article will be summarized in groups, based on the underlying mechanisms of increasing the effectiveness of therapeutic ionizing radiation.

### Targeting tumor hypoxia

Glioblastoma multiforme (GBM) is a tumor entity known for substantial hypoxic development. Furthermore, tumor hypoxia plays an important role as mediator of radioresistance, limiting the damage caused by ionizing irradiation based on reduction of oxidative stress as well as limitation of O_2_-mediated fixation of radiation-induced DNA damage. Several drugs aim to optimize perfusion and tissue oxygenation during radiotherapy (RT), trying to overcome this obstacle.

#### Nitroimidazoles

The earliest approach reviewed in this abstract was published in 1976: based on promising preclinical data [4], Urtasun et al. randomized a cohort of 31 patients with daily metronidazole during the 18-day course of ^60^Co-irradiation with opposing fields of two thirds of the brain versus radiation alone. Resulting in a statistically significant better time to progression (TTP) and overall survival (OS, median 26 vs 15 weeks) in the experimental treatment group, the authors attributed the effect to a delay of tumor regrowth resulting from “a higher cell inactivation of the radioresistant hypoxic cell population” [5]. Preclinical results had already shown metronidazole to be a potent radiosensitizer, especially in hypoxic cells based on further oxidization of radiation-induced oxidized lesions [4]. Subsequent trials were performed with its more potent successor misonidazole: in 1984, Fulton et al. followed up on the previous study combining this second-generation nitroimidazole with hyperfractionated radiotherapy (HFRT) and conventional fractions (CF). But while a multiple daily fractionated radiation therapy seemed beneficial, no significant improvement was obtained by the addition of the radiosensitizer [6]. A Vienna Study Group reported similar results the same year, showing no statistical significance concerning survival improvement [7]. Both study groups described low evidence of side effects during initial treatment, but after emerging evidence of accumulating toxicity in the form of peripheral neuropathy [8], studies shifted from using misonidazole to the third-generation drug etanidazole. Publications from Harvard Medical School reported feasibility of its use via continuous infusions during brachytherapy [9] as well as accelerated external beam radiotherapy (EBRT) for glioblastoma [10] and children’s brain stem glioma [11] with neuropathic symptoms in higher doses still defining the maximum tolerated dose. A follow-up on this trial by Chang et al. found the treatment to be well tolerated, but without improvement of survival compared to other treatment concepts [12]. Subsequently, after several trials without evidence of benefit regarding patient outcome, the use of nitroimidazoles in oncology shifted. With their affinity to hypoxic tissue proven beneficial in glioma research, nitroimidazole derivatives are being investigated in form of functional PET imaging for mapping tumor hypoxia with [F18]FETA or [F18]MISO imaging [13]. Clinical impact of this procedure remains to be demonstrated at this point. Table 1 summarizes the published trials evaluating nitroimidazoles as radiosensitizers.

**Table 1:** Trials evaluating nitroimidazoles as radiosensitizers, sorted by publication year

Author	Year	Agent	Dose	*n*	Radiation regimen	Results
Urtasun et al. [5]	1976	Metronidazole	6 g/m^2^ 3 times a week	36	3000 rads in 9 fractions (^60^Co), three times a week	Median TTP: 4.5 months Median OS: 26 weeks
Fulton et al. [6]	1984	Misonidazole	1.25 g/m^2^ 3 times weekly	128 (89)	58 Gy in 30 fractions (CF) vs 61.41 Gy in 69 fractions (3 × daily HFRT)	Median OS: 50 weeks (HFRT + MISO) vs 29 weeks (CF) vs 45 weeks (HFRT)
Stadler et al. [7]	1984	Misonidazole	2.1–2.7 g/m^2^ twice a week	45	66.5 Gy in 31 fractions	Median OS 13.8 months (vs 9.8 months RT alone)
Coleman et al. [9]	1992	Etanidazole	8–24 g/m^2^ continuous over 48–96 h	78 (42)	10 Gy/day brachytherapy	MTD of 96 h infusion is 23 g/m^2^
Riese et al. [10]	1994	Etanidazole	10–36 g/m^2^ continuously	70 (51)	40 Gy in 20 fractions bid, +20 Gy in 10 fractions vs 50 Gy ^125^I‑Brachy (4–5 fx)	MTD: 26 g/m^2^ for Brachytherapy, 34 g/m^2^ for External Beam Radiotherapy (EBRT)
Chang et al. [12]	1998	Etanidazole	10–36 g/m^2^ continuously	70 (51)	40 Gy in 20 fractions bid, +20 Gy in 10 fractions vs 50 Gy ^125^I‑Brachy (4–5 fx)	Median OS: 1.1 years (GBM)
Marcus et al. [11]	2003	Etanidazole	1.8–2.4 g/m^2^ daily	18	63–66 Gy in 42–44 fractions bid	MTD: 42 g/m^2^ in children with brain stem glioma

*n* = number of patients included (number of patients with glioblastoma in parentheses if multiple entities were included)

#### Hyperbaric oxygen

The concept of using hyperbaric oxygen to increase tissue oxygenation and overcome radioresistance has been explored for glioma treatment early on. In 1977, Chang et al. reported on a group of 80 patients randomized to standard radiation under either atmospheric air (*n* = 42) or hyperbaric oxygen (*n* = 38). With median survival of 38 weeks for the experimental group and 31 weeks for the control group, statistical significance was not obtained [14], but showed a trend encouraging further evaluation. Since setup difficulties arose from the use of hyperbaric oxygen during radiation treatment and preclinical data had proven feasibility of a sequential approach [15], subsequent studies focused on the effects of RT shortly after the use of hyperbaric oxygen. Kohshi et al. had already proven this concept to be applicable to patients with residual tumor after resection, combining hyperbaric oxygen with nitrosourea-based chemotherapy and external beam radiotherapy and found better treatment response when compared to a control group [16]. These results were later confirmed in other trials combining radiation and hyperbaric oxygen with nitrosourea-based chemotherapy [17–19]. Viability of RT after hyperbaric oxygen with overall low side effects was also proven for combined modality treatment alongside temozolomide (TMZ, the current therapy standard) throughout dose escalation to the surrounding edema [20] and even before fractionated stereotactic RT with a gamma unit in relapsed patients [21]. Although overall results were promising, especially regarding the added effectiveness when combined with TMZ, the lack of randomized controlled studies on larger cohorts and comparison to standard treatment means that the concept of hyperbaric oxygen as a way of overcoming tumor hypoxia remains under investigation [22]. Table 2 summarizes the published trials evaluating hyperbaric oxygen as a radiosensitizer.

**Table 2:** Trials evaluating hyperbaric oxygen as radiosensitizer, sorted by publication year

Author	Year	Agent	Dose	*n*	Radiation regimen	Results
Chang et al. [14]	1977	Hyperbaric oxygen	N/A	80	N/A	18 months survival: 28% (vs 10% atmospheric air) Median OS: 38 weeks (vs 31 weeks)
Kohshi et al. [16]	1996	Hyperbaric oxygen	60 min of 100% O_2_, 2.5 atm	21 (12)	50–71 Gy in 20–30 fractions	PR or CR in 100% (vs 33% atmospheric air)
		+ Nitrosourea	75 mg/m^2^ d1 + d36			
Beppu et al. [17]	2003	Hyperbaric oxygen	60 min of 100% O_2_, 2.8 atm	39 (29)	60 Gy in 30 fractions	Median TTP: 38 weeks (GBM)
		+ Nitrosourea	80 mg/m^2^ d1 + d36			
		+ IFN-beta	3 million IU/m^2^ 3 times a week			
Ogawa et al. [18]	2003	Hyperbaric oxygen	30–60 min of 100% O_2_, 2.8 atm	21 (15)	60 Gy in 30 fractions	1‑year OSR: 83% 2‑year OSR: 56% Median TTP: 15 months
		+ Procarbazine	90 mg/m^2^ d1–14			
		+ Nitrosourea	80 mg/m^2^ d1			
		+ Vincristine	0.5 mg/m^2^ d1 + d8			
Ogawa et al. [19]	2006	Hyperbaric oxygen	30–60 min of 100% O_2_, 2.8 atm	41 (31)	60 Gy in 30 fractions	Median TTP:12.3 months Median OS: 17.3 months
		+ Procarbazine	90 mg/m^2^ d1–14			
		+ Nitrosourea	80 mg/m^2^ d1			
		+ Vincristine	0.5 mg/m^2^ d1 + d8			
Kohshi et al. [21]	2007	Hyperbaric oxygen	60 min of 100% O_2_, 2.5 atm	25 (11)	Stereotactic gamma-radiation Median of 22 Gy in 8 fractions	Median OS: 11 months (GBM)
Yahara et al. [20]	2017	Hyperbaric oxygen	60–90 min of 100% O_2_, 2 atm	24	40 Gy in 20 fractions 16 Gy Boost in 8 fractions	Median OS: 22.1 months 2‑year OSR: 46.5%
		+ TMZ	75 mg/m^2^ daily			

*n* = number of patients included (number of patients with glioblastoma in parentheses if multiple entities were included)

#### Nicotinamide and carbogen

A similar approach to the use of hyperbaric oxygen is the breathing of carbogen during RT, resulting in higher solution of oxygen in blood plasma, leading to higher tissue oxygenation. This concept is often combined with oral intake of nicotinamide for better tumor perfusion by reducing the obturation of supplying blood vessels which results in a further decrease of tumor hypoxia [23]. Van der Maazen et al. first explored this concept in 1994 in a trial of 16 glioma patients and found results comparable to historical control cohorts, but unexpectedly high acute liver and subacute neurological side effects [24]. Pickles et al. obtained similar results where 50% of the patients developed grade 3 liver toxicity [25]. This led the authors to the presumption that the combination of nicotinamide, carbogen and radiation for glioma was to be used with caution, if at all. This was supported by the findings of several other small cohort studies [26, 27]. The EORTC 22933 trial evaluated the same concept on a larger scale, scrutinizing the different modalities, comparing accelerated 60 Gy RT (2 × 1.5 Gy daily) with nicotinamide, carbogen or both in a total of 107 patients. It found treatment arms including nicotinamide showing higher rates of side effects and therapy interruption without any evidence of benefit concerning median survival in any treatment group (10.1 months in RT + carbogen, 9.7 months in RT + nicotinamide, and 11.1 months in RT + both) when compared to standard treatment [28]. The concept was further challenged by Hulshof et al. who demonstrated no benefit in tumor or brain perfusion through the combination of both agents in a 99mTc-HMPAO SPECT study [29] and furthermore showed that treatment outcome could not be improved by the addition of intra-arterial cerebral chemotherapy with nimustine (ACNU) [23].

As a result, with overall low evidence of any treatment benefit, but consistent reports of high treatment toxicity, the concept of improving tumor oxygenation by combining oral nicotinamide and carbogen-breathing was abandoned. Table 3 summarizes the published trials evaluating nicotinamide and carbogen as radiosensitizers.

**Table 3:** Trials evaluating nicotinamide and carbogen as radiosensitizers, sorted by publication year

Author	Year	Agent	Dose	*n*	Radiation regimen	Results
Van der Maazen et al. [24]	1995	Carbogen Nicotinamide	5 min pre + during RT 6 g	16 (14)	50 Gy in 25 fractions	Median OS: 233 days High hepatic and neurotoxicity
Pickles et al. [25]	1996	Carbogen Nicotinamide	5–15 min pre RT 6 g, later 80 mg/kg	19	54 Gy in 30 fractions Later 50.1 in 30 fractions	Median OS: 8.5 months High hepatic toxicity
Fatigante et al. [26]	1997	Carbogen Nicotinamide	10 min pre + during RT 4 g + 2 g daily	36	60 Gy in 40 fractions bid	Median OS: 10 months High gastric and hepatic toxicity
Lambin et al. [27]	1997	Carbogen Nicotinamide	5 min pre + during RT 6 g	12	60 Gy in 30 fractions tid	Median OS: 7.2 months High hepatic and neurotoxicity
Miralbell et al. [28]	1999	Carbogen (C) Nicotinamide (N)	5 min pre + during RT 85 mg/kg	115	60 Gy in 40 fractions bid	Median OS: 11.1 months (RT + C + N) vs 10.1 months (RT + C) vs 9.7 months (RT + N)
Simon et al. [23]	2003	Carbogen Nicotinamide + ACNU	5 min pre + during RT 85 mg/kg 100 mg/m^2^, 3 cycles	33	59.4 Gy in 33 fractions	Median OS: 36.7 weeks High gastric toxicity

*n* = number of patients included (number of patients with glioblastoma in parentheses if multiple entities were included)

#### Tipifarnib

Tipifarnib is a farnesyltransferase inhibitor with the potential of increasing radiosensitivity by blocking activity of the RAS- and RhoB-oncogen pathways while also reducing tumor hypoxia by controlling MMP2 expression [30, 31]. After Cloughsey et al. reported evidence of activity of the drug in recurrent glioma [32], several studies combined tipifarnib with radiation therapy to exploit this mechanism. A 2007 phase I trial demonstrated good tolerance of the concept when accompanied by 60 Gy conventionally fractionated radiotherapy (CFRT), evaluating a maximally tolerated dose (MTD) of 200 mg/day [31]. This was subsequently challenged by higher doses: Lustig et al. reported on the use of tipifarnib in 28 patients with residual tumor, depending on antiseizure comedication, but found no signs of measurable responses in monthly MRIs and no benefit in overall survival [33]. A later study also included concomitant therapy with temozolomide (TMZ) without dose-limiting effects and acceptable results in short-term follow-up [34]. These positive results were confirmed by Ducassou et al. who followed up on their 2007 phase I trial [31] with median OS of 80.3 weeks and TTP of 23.1 weeks in 27 patients [35]. Since then, no further investigations of this double effective farnesyltransferase inhibitor have been made in glioma research. More recent approaches investigating the use of tipifarnib in combination with the multikinase inhibitor sorafenib (without the addition of RT) had to be stopped prematurely before finding a MTD because of severe side effects [36]. Table 4 summarizes the published trials evaluating tipifarnib as a radiosensitizer.

**Table 4:** Trials evaluating tipifarnib as radiosensitizer, sorted by publication year

Author	Year	Agent	Dose	*n*	Radiation regimen	Results
Moyal et al. [31]	2007	Tipifarnib	1 week before, during and after RT, starting at 200 mg/day	13	60 Gy in 30 fractions	200 mg/day tipifarnib is well tolerated Median OS: 12 months
Lustig et al. [33]	2008	Tipifarnib	300 or 600 mg bid, 3 weeks on, 1 week off Three cycles	28	60 Gy in 30 fractions	Median OS: 234.5 days No measurable response or improvement
Nghiemphu et al. [34]	2011	Tipifarnib	5–9 days pre and during RT 3 weeks on, 1 week off	51	60 Gy in 30 fractions	MTD: 300 mg bid Tolerated with concurrent TMZ
		+ TMZ	75 mg/m^2^ (partially)			
Ducassou et al. [35]	2013	Tipifarnib	100 mg bid 1 week before and during RT	27	60 Gy in 30 fractions	Median TTP: 23.1 weeks Median OS: 80.3 weeks

*n* = number of patients included (number of patients with glioblastoma in parentheses if multiple entities were included)

#### Efaproxiral

Efaproxiral is a synthetic allosteric hemoglobin-modifier that enhances tissue oxygenation in hypoxic areas by reducing the oxygen binding affinity through noncovalent bonds to hemoglobin. Kleinberg et al. reported on a phase I trial with 19 patients, showing good tolerance of combining the drug (100 mg/kg intravenous application directly before daily RT) with 60 Gy irradiation [37]. Following up on this with a phase II trial in 2002 where 50 patients enrolled, they found a median OS of 12.3 months and grade 2 toxicity of 24% [38]. Since this did not mark an improvement when compared to other combined modality treatments, this concept has not been explored further. Table 5 summarizes the published trials evaluating efaproxiral as a radiosensitizer.

**Table 5:** Trials evaluating efaproxiral as radiosensitizer, sorted by publication year

Author	Year	Agent	Dose	*n*	Radiation regimen	Results
Kleinberg et al. [37]	1999	Efaproxiral	100 mg/kg iv over 1 h before RT	19	60 Gy in 30 fractions	Treatment was well tolerated Increased oxygen unloading
Kleinberg et al. [38]	2002	Efaproxiral	100 mg/kg iv over 30 min before RT	50	60 Gy in 30 fractions	Median OS: 12.3 months

*n* = number of patients included (number of patients with glioblastoma in parentheses if multiple entities were included)

#### Tirapazamine

Tirapazamine is a benzotriazine compound that can be reduced to hydroxy radicals in hypoxic cells. Its combination with CFRT was evaluated in 2002 in a single phase II study by Del Rowe et al. with 124 patients [39]. A statistically significant benefit in overall survival was not discovered with median survival varying from 1.3 to 27.4 months in three different classes (divided according to patient and tumor characteristics to achieve comparability with a homogenous standard population of the model based on a RTOG database of 1500 cases). Median overall survival (10.8 months vs 9.5 months) as well as treatment tolerance was better in lower drug levels (159 vs 260 mg/m^2^ per infusion). It is noticeable that the treatment was comparable to combination treatment of 60 Gy RT with nitrosourea-based chemotherapy in the matched analysis. Since then, several trials have explored the use of tirapazamine in other tumor entities such as head and neck as well as gynecological cancers, but no further studies included glioma patients. Table 6 summarizes the published trial evaluating tirapazamine as a radiosensitizer.

**Table 6:** Trials evaluating tirapazamine as radiosensitizer

Author	Year	Agent	Dose	*n*	Radiation regime	Results
Del Rowe et al. [39]	2000	Tirapazamine	159 or 260 mg/m^2^ 3 × a week, 12 × overall	124	60 Gy in 30 fractions	Median OS: 10.8 months (159 mg/m^2^) vs 9.5 months (260 mg/m^2^)

*n* = number of patients included (number of patients with glioblastoma in parentheses if multiple entities were included)

### Interfering with repair of radiation-induced damage

Cytotoxic properties of ionizing radiation are diverse and can be subsumed into direct and indirect effects, leading to damage in cell components or in the DNA itself. Several radiosensitizers aim to stabilize the different types of damage (especially DNA damage) by preventing or interfering with cellular repair mechanisms.

#### Halogenated pyrimidines

The halogenated pyrimidines bromodeoxyuridine (BUdR) and iododeoxyuridine (IUdR) resemble the chemotherapeutic family of antimetabolites: after incorporation, dividing cells use them as a substitute for thymine in DNA synthesis or repair, resulting in higher vulnerability of cells with high mitotic index to primary or secondary radiation-induced DNA damage, such as single- or double-strand breaks or secondary damage from free radicals [40, 41]. To achieve this, a continuous supply of the drug with long-term intravenous infusion is needed. The search for optimal application schemes is a key factor in all research regarding this matter. The first approaches of Jackson et al. in 1987 which combined BUdR with conventionally fractionated and IUdR with hyperfractionated RT found no major differences between the two agents regarding survival (which was comparable to other combined-modality treatments), but higher incidences of phototoxicity and dermatologic side effects in short infusion regimens of BUdR (12 h infusions) [42]. In long-term infusion regimens (24 h infusions), hematological side effects were the main dose-limiting factor, as they were for treatment with IUdR as well. Further investigation of the use of BUdR by Matsutani et al. found similar results in 1988 [43], whereas Greenberg et al. assayed means to reduce systemic affection of the treatment. By direct intra-arterial infusion to the carotid arteries via infusion pump, the group aimed to prevent myelosuppression and skin reactions with lower drug doses and more direct delivery to the tumor site [44]. Overall toxicity was reduced and survival in the small cohort (18 patients, 15 with GBM) was better than previous results. Following them, the concept of intra-arterial infusion was further tested by Hegarty et al. with similar results [45] and evaluated concerning feasibility of co-administration of 5‑fluorouracil (5-FU) for further radiosensitization [46]. But since none of the mentioned studies generated OS improvement when compared to other combined-modality approaches, the increased risk of long-term intra-arterial infusion over the long course of radiation treatment for malignant glioma was not deemed suitable. Meanwhile, other groups further investigated intravenous concepts, focusing on finding the optimal duration for drug administration [47] as well as possible benefits when using hyperfractionated RT [48, 49]. Neither of the trials revealed clear evidence of a survival benefit and while IUdR had shown to be less photosensitizing [42, 48], most investigators reported high incidence of hematological toxicity regardless of the agent. Especially in combination with systemic chemotherapy like 5‑FU [50] or the PCV scheme [51], the use of iv radiosensitization with halogenated pyrimidines caused increased amounts of grade III and IV toxicity, last-mentioned in a large phase III trial (RTOG 9404) on patients with anaplastic astrocytoma in 2004, which also concluded a lack of survival benefit [52]. With emerging relevance of combination treatment of RT and temozolomide or nitrosoureas (both also having accompanying potential for myelosuppression and lymphopenia, a side effect with unclear relevance concerning treatment outcome [53]), the use of halogenated pyrimidines as radiosensitizers for glioma treatment did not prove to be profitable enough and was abandoned. Table 7 summarizes the published trials evaluating halogenated pyrimidines as radiosensitizers.

**Table 7:** Trials evaluating halogenated pyrimidines as radiosensitizers, sorted by publication year

Author	Year	Agent	Dose	*n*	Radiation regimen	Results
Jackson et al. [42]	1987	BUdR	650 mg/m^2^/day as 12 h or 24 h-iv, 2 × 14 days	60 (50)	65–70 Gy in 35 fractions (BUdR)	Median OS: 13 months IUdR vs BUdR: no survival difference
		IUdR	1000 mg/m^2^/day as 12 h or 24 h-iv, 2 × 14 days		45 Gy in 30 fractions bid +25 Gy Boost in 20 fractions bid (IUdR)	
Matsutani et al. [43]	1988	BUdR	800–1000 mg/m^2^/day for 5 days a week	23 (7)	50–60 Gy in 25–30 fractions	Median TTP (GBM): 37 weeks
Greenberg et al. [44]	1988	BUdR	400 mg/m^2^/day as 24 h-iv for 8 weeks	18 (15)	59.4 Gy in 33 fractions	Median OS: 22 months
Hegarty et al. [45]	1990	BUdR	400–600 mg/m^2^/day as 24 h-iv, 8.5 weeks	23 (18)	59.4 Gy in 33 fractions	Median OS: 20 months
Phillips et al. [51]	1991	BUdR	800 mg/m^2^/day as 24 h-iv for 4 days a week	160	60 Gy in 30 fractions	Median OS: 55.7 weeks Median TTP: 34.5 weeks
Goffman et al. [48]	1992	IUdR	1000 mg/m^2^/day as 12 h or 24 h-iv, 2 × 14 days	45	45 Gy in 30 fractions bid +25–30 Gy boost in 20 fractions bid	No significant benefit of IUdR Median OS: 11 months
Vokes et al. [50]	1993	IUdR	125–500 mg/m^2^/day as 24 h-iv, 2 × 5 days	15 (11)	65 Gy in 36 fractions	Significant systemic toxicity when combined with 5‑FU and HU
		+ 5-FU	300 mg/m^2^/day, 5 days			
		+ Hydroxyurea	500 mg tid, 11 doses			
Urtasun et al. [47]	1993	IUdR	1000 mg/m^2^/day as 24 h-iv, 48 h-iv or 96 h-iv	79 (56)	60.16 Gy in 32 fractions	Median OS: 13.4 months for 96 h-iv (vs 10.5 months for 48 h-iv vs 11 months for 24 h-iv)
Greenberg et al. [46]	1994	BUdR	400 mg/m^2^/day 24 h-iv	62 (58)	59.4 Gy in 33 fractions	Median OS: 18 months Co-delivery with 5‑FU tolerable
		+ 5-FU	5 mg/m^2^/day iv for 8.5 weeks			
Groves et al. [49]	1999	BUdR	2.1 g/m^2^/day as 24 h-iv, 2 × 4 days	88	55.5–57 Gy in 30 fractions tid, one week on, one week off	Median OS: 50 weeks, Median TTP: 28.5 weeks High derma- and hematological toxicity

*n* = number of patients included (number of patients with glioblastoma in parentheses if multiple entities were included)

#### PARP inhibitors

The poly(ADP-ribose) polymerase (PARP) proteins are intracellular mediators for discovery and management of DNA damage by activating pathways of homologous recombination (for repair of single-strand breaks) and nonhomologous end-joining (for repair of double-strand breaks) [54, 55]. The inhibition of these signaling proteins via PARP inhibitors (PARPi) is being reviewed for potential radiosensitizing as well as chemosensitizing properties. Based on the verification of PARP activity in human glioblastoma by Galia et al. in 2012 [56], several studies have evaluated these effects. Su et al. reported on a series of 29 children with recurrent tumors of the central nervous system (three with GBM), treated with the PARP inhibitor veliparib in combination with TMZ and (mostly, 90%) RT. With overall acceptable tolerability of the combined modality treatment, but increased instances of hematotoxicity, the group concluded with an optimistic subsumption of the combination [54]. A follow-up study on children with diffuse pontine glioma was initiated but failed to improve survival [57]. In adult patients, Robins et al. evaluated veliparib as an addition to TMZ chemotherapy (for chemosensitization) in patients with recurrent glioblastoma, and experienced heightened accounts of myelosuppression [58] as well. Hanna et al. found similar results regarding the PARP inhibitor olaparib in the OPARATIC trial [59]. However, their evaluation of tissue penetration and radiation response in vitro found activity of the agent in radiosensitizing doses in glioma tissue. Nevertheless, a trial by Sim et al. on 125 patients where veliparib was combined with glioblastoma treatment, consisting of 60 Gy irradiation and sequential temozolomide did not show survival benefit (but treatment was tolerated well) [60], while another trial using olaparib instead of veliparib (OLA-TMZ-RTE-01) is ongoing momentarily [61]. So, while the underlining mechanisms of PARP inhibition seem to be promising for glioma treatment, evidence of benefit regarding patient outcome has not yet been found. Table 8 summarizes the published trials evaluating PARP inhibitors as radiosensitizers.

**Table 8:** Trials evaluating PARP inhibitors as radiosensitizers, sorted by publication year

Author	Year	Agent	Dose	*n*	Radiation regimen	Results
Su et al. [54]	2014	Veliparib	20–30 mg/m^2^/dose bid	31 (3)	N/A	Overall tolerable combination with TMZ, increased hematotoxicity
		+ TMZ (partially)	180 mg/m^2^/day			
Lesueur et al. [61]	2019	Olaparib	50–200 mg bid	79	60 Gy in 30 fractions	Trial ongoing, evaluating feasibility and outcome of RT + TMZ + PARPi
		+TMZ	75 mg/m^2^			
Sim et al. [60]	2021	Veliparib	200 mg bid	125	60 Gy in 30 fractions	Median OS: 12.7 (RT + TMZ + PARPi) vs 12.8 months (RT + TMZ alone) Feasibility of RT + adjuvant TMZ + PARPi

*n* = number of patients included (number of patients with glioblastoma in parentheses if multiple entities were included)

#### Motexafin gadolinium (MGd)

Motexafin gadolinium (MGd) is a compound of gadolinium and an expanded porphyrin, resulting in texaphyrin. Pharmaceuticals of this class have been investigated as radiosensitizers in combination with cerebral irradiation in several tumor entities [62–66], relying on the additional generation of reactive oxygen species and interference with repair mechanisms of radiation-induced damage which lead to increased cell death [67, 68]. Wu et al. demonstrated promising data for use in glioma treatment in 2006: MGd uptake in human GBM was proven in vivo without penetration of the drug into areas with intact blood–brain barrier, potentially increasing RT effectiveness in tumor tissue, while having little to no impact on normal tissue complications [69]. Based on this, a phase I trial was established which showed good tolerance of the concept when combined with standard radiation treatment (59.4 Gy in 33 fractions) and a trend towards a survival benefit (16.1 months compared to 11.8 months in a matched analysis with the RTOG database) [67]. But a follow-up phase II trial by Bachman et al. in 2015, which combined daily MGd before RT with standard TMZ chemotherapy, did not increase survival benefit when compared to other combined modality treatment (median OS: 15.6 months) [70]. A possible reason for the lack of benefit might be the reduced tissue penetration of MGd in border regions of the tumor tissue, which are precisely the areas with high risk for relapse [69]. Table 9 summarizes the published trials evaluating motexafin gadolinium as a radiosensitizer.

**Table 9:** Trials evaluating motexafin gadolinium (MGd) as a radiosensitizer, sorted by publication year

Author	Year	Agent	Dose	*n*	Radiation regimen	Results
Ford et al. [67]	2007	Motexafin Gadolinium	10–22 × 4–5.2 mg/kg/day	33	59.4 Gy in 33 fractions	MTD: 5 mg/kg/day Median OS: 16.1 months
Bachman et al. [70]	2015	Motexafin Gadolinium	3–5 mg/kg daily pre RT	118	60 Gy in 30 fractions	MTD MGd: 5 mg/kg/day Median OS: 15.6 months RT + MGd + TMZ was well tolerated
		+ TMZ	75 mg/m^2^/day			

*n* = number of patients included (number of patients with glioblastoma in parentheses if multiple entities were included)

#### Difluoromethylornithine (DFMO)

DFMO is a polyamine synthesis inhibitor which has been used in different contexts as a radiosensitizer [71, 72]. While the exact mechanism remains not fully understood [71], the reduction of cellular polyamine seems to prevent DNA stabilization, leading to radiosensitization by reduced recovery of damage inflicted by ionizing radiation [73]. After promising preclinical [71] and clinical [72] results in other tumor entities, Prados et al. enrolled 231 patients in a large phase III trial to investigate the benefit of DFMO when added to either conventionally (CFRT) or hyperfractionated (HFRT) radiotherapy in patients with newly diagnosed glioblastoma [73]. While overall little side effects were reported, DFMO arms showed increased low-grade toxicity, a few cases of hearing impairment, and did not conclude a statistically significant impact on progression-free or overall survival. Hyperfractionated radiotherapy also did not show an advantage over conventionally fractionated RT; therefore, the investigators concluded with the recommendation of neither of the two evaluated concepts (addition of DFMO and HFRT). Table 10 summarizes the published trial evaluating DFMO as a radiosensitizer.

**Table 10:** Trials evaluating difluoromethylornithine (DFMO) as a radiosensitizer, sorted by publication year

Author	Year	Agent	Dose	*n*	Radiation regimen	Results
Prados et al. [73]	2001	DFMO	1.8 gm/m^2^ tid	231	70.4 Gy in 44 fractions bid (HFRT) vs 59.4 Gy in 33 fractions (CFRT)	Median OS: HFRT 40 weeks, HFRT + DFMO: 42 weeks CFRT: 37 weeks CFRT + DFMO: 44 weeks

*n* = number of patients included (number of patients with glioblastoma in parentheses if multiple entities were included)

### Enhancing apoptotic pathways

Scientific as well as technical advances have and will allow for a better, more detailed understanding of cellular mechanisms of interaction and communication as well as invasion strategies into cells and their internal signaling pathways. This leads to an increase of possible targets in modern oncology with more starting points for targeted therapies, but also agents of radiosensitization, leading to apoptosis in an array of interactions.

#### Interferon-alpha2a

Recombinant interferons are used in cancer treatment as immunomodulators with antiproliferative and antiangiogenetic properties [74]. Via pathways of enhancing p53, they also yield radiosensitizing potential by increasing the amount of cell death by radiation-induced apoptosis [74]. Dillmann et al. explored this concept in 1995 in a phase I/II trial with little toxicity [75], combined with conventionally fractionated radiotherapy for patients with newly diagnosed glioblastoma. A follow-up trial also explored the addition of cis-retinoic acid (CRA) to the treatment, which had shown an additive effect in combination with interferon-alpha in previous studies [76]. Again, feasibility of the concept was proven, but without establishing a benefit in survival when compared to other treatments [77]. Table 11 summarizes the published trials evaluating interferon-alpha2a as a radiosensitizer.

**Table 11:** Trials evaluating interferon-alpha2a as a radiosensitizer, sorted by publication year

Author	Year	Agent	Dose	*n*	Radiation regimen	Results
Dillman et al. [75]	1995	IFN-alpha2a	3–5 million IU sc. for 3 days/week	19 (12)	59.4 Gy in 33 fractions	Median OS: 7.4 months
Dillman et al. [77]	2001	IFN-alpha2a	3–6 million IU s.c. qid for 3 days/week	40 (36)	59.4 Gy in 33 fractions	Median OS: 9.3 months
		CRA	1 mg/kg qid for 5 days/weeks			

*n* = number of patients included (number of patients with glioblastoma in parentheses if multiple entities were included)

#### Lovastatin

Lovastatin is a lipid-lowering drug that also influences radiation sensibility in multiple ways: by interfering with signaling pathways leading to apoptosis (such as p53), sensibility to radiation-induced cell damage was found to be increased [78], while also having protective capabilities in endothelial tissue without impairing induction of double-strand breaks [79]. A single trial in 1998 evaluated the benefit of cotreatment with ionizing radiation (without specification regarding dosage of the agent or radiation). The overall treatment was tolerated well, but the trial did not deliver long-term results or follow-up investigations [80]. No further trials concerning the use of statins as radiosensitizers in glioma treatment have been published since. An analysis of two glioblastoma trials in 2018 did not detect an impact of comedication with statins (among others) concerning patient outcome [81]. Table 12 summarizes the published trial evaluating lovastatin as a radiosensitizer.

**Table 12:** Trials evaluating lovastatin as a radiosensitizer, sorted by publication year

Author	Year	Agent	Dose	*n*	Radiation regimen	Results
Larner et al. [80]	1998	Lovastatin	N/A	18	N/A	Combination with RT is well tolerated

*n* = number of patients included (number of patients with glioblastoma in parentheses if multiple entities were included)

#### Boron neutron capture

While it is hardly comparable to standard photon irradiation, the premise of radiosensitization in neutron irradiation via boron neutron capture therapy (BNCT) shares similarities. Infusions with stabilized boron-10 (in the form of *p*-boronophenylalanine [BPA] or sodium borocaptate [BSH]) are used prior to thermal or epithermal neutron irradiation, leading to higher energy transfer by producing high linear energy transfer particles with short range which causes subsequent local cell death. With preferred uptake in tumor tissue, the treatment aims to achieve high toxicity in tumor cells with minimal risk of damaging surrounding tissue [82]. Beginning in 1994, several study groups have evaluated this concept as an alternative treatment method for malignant glioma [82–85], optimizing the concepts of dose delivery and monitoring [86–89] as well as exploring concepts of intraoperative treatment [90], combination of different boron sources (BPA + BSH, [91, 92]), different types of radiation (neutrons + photons, [93, 94]) and palliative approaches [95]. But while several studies showed promising results [89–92, 96, 97] as well as histopathologic proof of treatment response [98, 99], the benefit did not exceed standard therapy with photon irradiation and concurrent chemotherapy with TMZ [100]. While evidence seems to indicate a possible advantage for patients with unmethylated MGMT promotor [101, 102], the overall small number of participants in studies utilizing the concept of boron neutron capture therapy does not yet allow a clear verdict on the concept. Further randomized trials with larger patient numbers are needed [103], but the complexity of the treatment as well as the required infrastructure seem to be considerable roadblocks in this regard [100]. The concept of particle irradiation for glioblastoma patients is also a present topic in contemporary research [104]. Table 13 summarizes the published trials evaluating BNCT in glioblastoma treatment.

**Table 13:** Trials evaluating boron neutron capture therapy (BNCT) sorted by publication year

Author	Year	Agent	Dose	*n*	Radiation regimen	Results
Coderre et al. [84]	1997	BPA	130–250 mg/kg	18	Maximum of 52.6 ± 4.9 Gy-Eq Minimum of 25.2 ± 4.2 Gy-Eq to the tumor	Feasibility of the concept, no adverse events
Takagaki et al. [85]	1997	BSH	20 mg/kg	11	N/A	2‑year OSR: 50%
Chadha et al. [86]	1998	BPA	250 mg/kg	10	Minimum of 20 to 32.3 Gy-Eq to the tumor	Median OS: 13.5 months
Palmer et al. [83]	2002	BPA	250–350 mg/kg	22	8.8–14.2 Gy-Eq in 1–2 fractions	Feasibility of the concept Mean tumor dose: 25.7 RBE Gy
Capala et al. [87]	2003	BPA	900 mg/kg	17	Maximum of 8.0–15.5 Gy-Eq Average dose 3.3–6.1 Gy-Eq to the brain	No severe acute toxicities
Diaz et al. [96]	2003	BPA	250–330 mg/kg	53	Total of 8.9–15.9 Gy-Eq delivered in 1–3 fields	Feasibility of the concept
Kageji et al. [88]	2004	BSH	N/A	18 (16)	Maximum of 15–18 Gy-Eq to the tumor	Maximum vascular dose should be below 12 Gy
Kiger et al. [89]	2004	BPA	14 g/m^2^	6	Maximum 7–7.7 Gy-Eq to the whole brain in two fractions	Median tumor dose: 57.8 RBE Gy
Yamamoto et al. [90]	2004	BSH	100 mg/kg	9 (5)	Maximum vascular dose 10.8 Gy-Eq, 1 intraoperative fraction	Median OS: 23.2 months (GBM)
Miyatake et al. [91]	2005	BSH & BPA	5 g 250 mg/kg	13 (10)	Maximum of 13 Gy-Eq to the normal brain	Mean volumetric reduction: 46.4%
Stenstam et al. [98]	2007	BPA	900 mg/kg	7	Minimum of 36.9 Gy-Eq to the tumor (mean)	Postmortem whole brain slices showed local control in all cases
Henriksson et al. [100]	2008	BPA	900 mg/kg	30	Average 3.2–6.1 Gy-Eq to normal brain, minimum of 15.4–54.3 Gy-Eq to the tumor	Median TTP: 5.8 months Median OS: 14.2 months
Miyatake et al. [92]	2009	BSH & BPA	100 mg/kg 250–600 mg/kg	22 (19)	Maximum of 13 Gy-Eq to the normal brain	Median OS: 9.6 months (rGBM)
Kawabata et al. [93]	2009	BSH & BPA	100 mg/kg 250–700 mg/kg	21	Maximum of 13–15 Gy-Eq to the normal brain, partially followed by 20–30 Gy photons (11)	Median OS: 15.6 months vs 23.5 months in combination with photons
Aiyama et al. [95]	2011	BPA	250 mg/kg	2 (1)	Maximum of 12 Gy-Eq to the normal brain	No adverse events
Kankaanranta et al. [97]	2011	BPA	350–450 mg/kg	22 (20)	Maximum average 6 Gy-Eq to normal brain, maximum peak of 8 Gy	MDT: 400 mg/kg Median OS: 7 months (rGBM)

*n* = number of patients included (number of patients with glioblastoma in parentheses if multiple entities were included)

#### 5-Aminolevolinic acid

5‑Aminolevolinic acid (5-ALA) is a ketone carbon amino acid with several interesting capabilities in glioma treatment. So far, it is mainly used in resective surgery to increase the extent of tumor resection via visualizing tissue infiltration. Oral intake of pharmacological 5‑ALA leads to an accumulation in glioma tissue and enzymatic transformation to protoporphyrin IX (PPIX), allowing for fluorescence-guided resection which has proven to increase progression-free survival after surgery [105]. Furthermore, stimulation of PPIX-enriched tissue with light of a certain wavelength and energy leads to induction of cell death, making the treatment of glioblastoma patients with 5‑ALA-based photodynamic therapy (PDT) a valuable concept in cases with reduced resective potential [106]. In this analysis, multiple trials that were assessed as not fitting the context of the matter at hand directly were covering PDT, possibly due to the similarity of photosensitization to radiosensitization. But while the impact of photodynamic and surgical therapy with 5‑ALA is covered elsewhere [107, 108], there is also evidence of radiosensitizing capability. In vitro and rodent models have shown increased cell death induced by mitochondrial oxidative stress and production of reactive oxygen species after photon irradiation [109–111]. Additional experiments on other cell lines have proven this effect to also occur under high energy photon beam irradiation with 15 MV, as used in modern radiation oncology [112, 113], but trials involving human patients are still missing.

### Radiosensitizing effects of chemotherapeutic drugs in glioblastoma treatment

For the sake of completion, it should be stated that several chemotherapeutic drugs also yield radiosensitizing potential. However, since their use in glioblastoma treatment derives from their cytoreductive nature, and the accompanying increase of effectiveness of RT is more of a side effect, we will not fully cover the extent of reported trials, but address the underlining mechanisms of radiosensitization concerning the relevant agents of systemic glioma therapy.

#### Temozolomide

As an alkylating chemotherapeutic drug, temozolomide has become standard treatment in systemic adjuvant therapy for glioblastoma multiforme, based on the trial by Stupp et al. [2]. By methylating radiation-induced lesions such as double-strand breaks, preferably on the O6-atom of adenine, temozolomide can stabilize is damage, leading to increased effectiveness of RT concomitant to TMZ application [114]. Unfortunately, tumors expressing O6-methylguanine DNA methyltransferase (MGMT) show increased potential of repairing these lesions, which leads to a decreased effectiveness of TMZ therapy in patients with nonmethylated MGMT promotor [115], resulting in poorer prognosis [116]. Scientific studies currently try to overcome these limitations for example by using a compound-drug to increase the alkylating effects in MGMT-expressing tumor tissue [117].

#### Nitrosoureas

Nitrosoureas like ACNU, BCyNU and CCNU are alkylating substances with abilities of cross-linking DNA, working cell-cycle dependent and independent [118]. Herein, the majority of activity seems to happen in the late S phase, the most radioresistant phase of the cell cycle [119, 120], making nitrosoureas a valuable asset to radiation therapy by targeting resistant cells in recurrent glioma as well as in other glioma entities like oligodendroglioma, e.g., in combinations like the PCV scheme [118, 121, 122]. A novel combination of CCNU and TMZ concurrent with RT evaluated in the CeTeG/NOA-09 trial did show improved overall survival in patients with newly diagnosed MGMT-methylated glioblastoma multiforme [3], but since possible downsides regarding effectiveness of lomustine in subsequent recurrence are being discussed, the use of the combination is still limited [123]. The additional intrinsic effect of nitrosoureas of radiosensitization by inhibition of the glutathione reductase [119] in this combination is not yet fully understood.

#### Procarbazine

Procarbazine is an alkylating chemotherapeutic drug, used as part of the PCV-combination scheme in glioma treatment. But while the accompanying vincristine does not seem to have radiosensitizing capabilities [124], such potential was demonstrated for procarbazine in hypoxic cells, based on preclinical data because of the embodied redox potential of its structure [125]. Even though the combination of lomustine (CCNU) and vincristine is highly beneficial in grade 2 and 3 glioma, especially regarding the oligodendroglial subtype [126, 127], its use in the treatment of glioblastoma seems to be of limited effectiveness when compared to other regimens [3, 121, 122, 128].

#### Taxanes

Targeting the assembly of the mitotic spindle, the taxane family of chemotherapeutic drugs disrupts the cell cycle, resulting in a G2/M cell cycle arrest. This phase has been proven to show increased sensitivity to ionizing radiation, resulting in taxanes to be valuable radiosensitizers [129]. Especially paclitaxel has been studied extensively for glioblastoma in this regard [130–133]. At first, it showed promising results, for example in combination with high fraction doses [134], as an alternative treatment for older patients or with reduced performance status [135], but overall, it showed little benefit when compared to other regimens. PPX, a conjugate of paclitaxel and poly-L-glutamic acid that showed increased radiosensitization in rodent models [136], also initially resulted in increased progression-free survival when combined with radiation and temozolomide [137]. But a large follow-up study (BrUOG 244) could not reproduce these benefits [138] and therefore, the concept of taxanes as a radiosensitizers in glioma therapy has been abandoned for the time being.

#### 5-Fluorouracil (5-FU)/capecitabine

5‑Fluorouracil and its oral prodrug capecitabine, as members of the family of antimetabolites, increase effectiveness of ionizing radiation when administered simultaneously via several mechanisms. They target and kill radioresistant cells in the S phase, similar to how nitrosoureas [139] do, and reduce the repair of induced DNA damage by blocking the synthesis of thymidine [140]. 5‑FU was used in glioma treatment in combination with several other chemotherapeutic agents [141–143] as well as radiosensitizers [46, 50], but did not reach standard therapy status. Modern research explored the continuous application of 5‑FU via locally applied microspheres, a new concept with potential benefits in glioma treatment with inherent radiosensitization [144, 145].

#### Gemcitabine

Gemcitabine is a deoxynucleoside analogue with radiosensitizing qualities deriving from several mechanisms of reducing DNA repair and lowering thresholds for apoptotic pathways and redistributing cells in the cell cycle [146, 147]. Since it is also capable of passing the blood–tumor barrier in human glioma [146] and its activity is observable in MGMT-methylated and -unmethylated tumors [148], combination therapy has been explored in several phase I and II trials but failed to improve survival outcome [148–152]. Contemporary research explores novel drug-conjugates and a different way of intratumoral delivery of gemcitabine as injectable hydrogel in preclinical glioma settings [153, 154].

#### Platinum derivates (cisplatin/carboplatin)

As alkylating chemotherapeutic agents, platinum derivates cisplatin and carboplatin can synergize with ionizing radiation via the inhibition of nonhomologous end joining which results in the stabilization of radiation-induced damage [155]. While the use of platinum-based therapy concurrent to radiation is a method of increasing therapeutic effectiveness in a variety of malignancies, combination schemes did not result in survival improvement for glioma patients. Moreover, their use was associated with increased treatment toxicity, limiting the applicable dose and therefore effectiveness [156, 157], possibly due to poor penetration of the blood–brain barrier [158]. Newer approaches try to circumvent this obstacle, for example with liposomal-coated drugs, but have not yet reached clinical testing [160–162]; this concept is also currently explored in similar circumstances with doxorubicin in different tumor entities [159].

#### Bevacizumab

Glioblastoma multiforme is a tumor entity with a high degree of vascular proliferation, a process reducing therapy effectiveness due to inadequate vascularization leading to tumor hypoxia and insufficient distribution of chemotherapeutic agents [163, 164]. As a monoclonal antibody to vascular endothelial growth factor A, bevacizumab presents a therapy challenging this mechanism by reducing tumor angiogenesis, thus increasing proper perfusion, and lowering hypoxia and therefore radioresistance [165]. Several phase II trials showed promising results [166, 167], but recent phase III trials did not find a survival benefit [168–170]. Hence, the role of anti-VEGF therapy in combined modality treatment remains unclear and its use remains restricted to second line treatment of recurrent glioma with high local variability regarding approval state for this indication [123, 171].

## Discussion

Despite major scientific efforts, the prognosis of patients with glioblastoma multiforme remains poor. The use of fluorescence-guided resection and the introduction of temozolomide as systemic treatment managed to achieve longer sustained survival, and radiation therapy plays a key role in postponing the seemingly inevitable relapse. Since tumor recurrence mostly occurs in areas bordering on the initial treatment site, the use of radiosensitizers seems to be a feasible option to optimize local control. Clinical trials have evaluated several different agents, aiming for increased patient outcome so far, capitalizing on different pathways of increasing the effectiveness on ionizing radiation. The reduction of tumor hypoxia with agents like nitroimidazoles or nicotinamide in combination with carbogen breathing peaked at a median OS of 13.8 months (misonidazole [7]) and 11.1 months (nicotinamide [28]) but showed high incidents of neuropathic or intestinal side effects. Other agents aimed to reduce DNA repair: halogenated pyrimidines achieved promising results in studies with mixed tumor grades (up to 22 months [44]) but the largest cohort of only glioblastoma patients did not verify a benefit (median OS 55.7 weeks [51]) and resulted in increased toxicity. Novel approaches in this strategy, like PARP inhibition or the use of compound-gadolinium also failed to show statistically significant benefit as an asset to the implemented Stupp regimen (MGd: 15.6 months [70], PARPi: 12.7 months [60]). A very interesting approach is the concept of BNCT, which has already shown some encouraging results (median OS of 23.2 months [90], 2‑year OSR of 50% [85]) but the complexity of the treatment results in overall low case numbers. Future studies with larger cohorts are needed to validate this promising data.

All in all, while the variety of methods and agents examined for radiosensitizing benefit in glioblastoma therapy is large, most substances either failed to improve survival when compared to standard treatment or lack validation via phase III trials with large cohorts. Thus, the combination of radiotherapy with concurrent and adjuvant temozolomide as introduced by Stupp et al. in 2005 (leading to a median overall survival of 14.6 months in the respective trial) remains the standard of care for glioblastoma and all future therapy approaches will be measured against it. Trials like Yahara et al. [20] demonstrated that the current standard of the RT + TMZ can be elevated even further when the regimen is complemented by another method of radiosensitization (in this case hyperbaric oxygen, leading to a median OS of 22.1 months), but again, further research and lager cohorts are needed.

However, while several chemotherapeutic drugs like temozolomide in the standard Stupp regimen or new combinations (e.g., with lomustine [3]) also capitalize on the inherent radiosensitizing effect of the compounds, most additional agents failed to improve glioma therapy. Obstacles often seemed to be penetration of the blood–brain barrier and tumor selectiveness. In this regard, the photosensitizing agent 5‑aminolevolinic acid has also presented radiosensitizing capabilities in preclinical trials. This substance has proven its benefit concerning accumulation in glioma tissue in the context of resective surgery and PDT, but clinical studies are yet to confirm the approach of combining it with ionizing radiation. A benefit of 5‑ALA is its sparing of normal brain tissue and selectiveness to glioma cells because of active uptake after passing the defective blood–brain barrier and diffusion with surrounding edema [108]. Limitations might derive from limited tissue penetration [172, 173], individual variations in the generation of the active substance PPIX [174] and uncertainties regarding toxicity of repeated administration of 5‑ALA (based on the lack of data). Whether this concept might result in a valuable new treatment strategy or in a dead end is uncertain at this point. Nevertheless, additional scientific effort is needed to design other substances capable of increasing the effectiveness of RT in glioblastoma with better tissue penetration and ideally greater radiosensitizing capabilities.

## Conclusion and outlook

Although initial results were often promising, the search for ways to improve survival rates for patients with glioblastoma multiforme via radiosensitization has mostly been unsuccessful. At our institution, we aim to further investigate the safety and effectiveness of 5‑ALA as a possible radiosensitizer in combination with standard ionizing irradiation for patients with glioblastoma. In addition, novel agents, drug-conjugates or alternative approaches of delivery or sensitization are still being explored [175]. Scientific effort regarding this topic still is far from complete.

## Abbreviations

ACNU: Nimustine
bid: *bis in die*, twice a day
BNCT: Boron neutron capture therapy
CCNU: Lomustine
CF: Conventional fractions
CFRT: Conventionally fractionated radiotherapy
CR: Complete response
CRA: Cis-retinoic acid
DFMO: Difluoromethylornithine
EBRT: External beam radiotherapy
GBM: Glioblastoma multiforme
HFRT: Hyperfractionated radiotherapy
iv: intravenous infusion
MGd: Motexafin gadolinium
MGMT: O6-methylguanine DNA methyltransferase
MTD: Maximum tolerated dose
N/A: Not available
OS: Overall survival
OSR: Overall survival rate
PARP: Poly(ADP-ribose) polymerase
PARPi: PARP inhibitor
PCV: Procarbazine + Lomustine + Vincristine combination chemotherapy
PR: Partial response
RT: Radiation therapy
sc: Subcutaneous injection
tid: *ter in die*, three times a day
TMZ: Temozolomide
TTP: Time to progression
5‑FU: 5‑Fluorouracil

## References

[1] Stummer W, Novotny A, Stepp H, Goetz C, Bise K, Reulen HJ (2000). Fluorescence-guided resection of glioblastoma multiforme by using 5-aminolevulinic acid-induced porphyrins: a prospective study in 52 consecutive patients. J Neurosurg.

[2] Stupp R, Mason WP, van den Bent MJ (2005). Radiotherapy plus concomitant and adjuvant temozolomide for glioblastoma. N Engl J Med.

[3] Herrlinger U, Tzaridis T, Mack F (2019). Lomustine-temozolomide combination therapy versus standard temozolomide therapy in patients with newly diagnosed glioblastoma with methylated MGMT promoter (CeTeG/NOA-09): a randomised, open-label, phase 3 trial. Lancet.

[4] Asquith JC, Foster JL, Wilson RL (1974). Metronidazole (‘Flagyl’). A radiosensitizer of hypoxic cells. Br J Radiol.

[5] Urtasun R, Band P, Chapman JD, Feldstein ML, Mielke B, Fryer C (1976). Radiation and high-dose metronidazole in supratentorial glioblastomas. N Engl J Med.

[6] Fulton DS, Urtasun RC, Shin KH (1984). Misonidazole combined with hyperfractionation in the management of malignant glioma. Int J Radiat Oncol Biol Phys.

[7] Stadler B, Karcher KH, Kogelnik HD, Szepesi T (1984). Misonidazole and irradiation in the treatment of high-grade astrocytomas: further report of the Vienna study group. Int J Radiat Oncol Biol Phys.

[8] Overgaard J (2011). Hypoxic modification of radiotherapy in squamous cell carcinoma of the head and neck—a systematic review and meta-analysis. Radiother Oncol.

[9] Coleman NC, Noll L, Riese N, Buswell L, Howes AE, Loeffler JS (1992). Final report of the phase I trial of continuous infusion etanidazole (SR 2508): a radiation therapy oncology group study. Int J Radiat Oncol Biol Phys.

[10] Riese NE, Loeffler JS, Wen P, Alexander E, Black PML, Coleman CN (1994). A phase I study of etanidazole and radiotherapy in malignant glioma. Int J Radiat Oncol Biol Phys.

[11] Marcus KJ, Dutton SC, Barnes P, Coleman CN, Pomeroy SL, Goumnerova L (2003). A phase I trial of etanidazole and hyperfractionated radiotherapy in children with diffuse brainstem glioma. Int J Radiat Oncol Biol Phys.

[12] Chang EL, Loeffler JS, Riese NE, Wen PY, Alexander E, Black PM (1998). Survival results from a phase I study of etanidazole (SR2508) and radiotherapy in patients with malignant glioma. Int J Radiat Oncol Biol Phys.

[13] Chakhoyan A, Guillamo JS, Collet S, Kauffmann F, Delcroix N, Lechapt-Zalcman E (2017). FMISO-PET-derived brain oxygen tension maps: application to glioblastoma and less aggressive gliomas. Sci Rep.

[14] Chang CH (1977). Hyperbaric oxygen and radiation therapy in the management of glioblastoma. Natl Cancer Inst Monogr.

[15] Beppu T, Kamada K, Yoshida Y, Arai H, Ogasawara K, Ogawa A (2002). Change of oxygen pressure in glioblastoma tissue under various conditions. J Neurooncol.

[16] Kohshi K, Kinoshita Y, Terashima H, Konda N, Yokota A, Soejima T (1996). Radiotherapy after hyperbaric oxygenation for malignant gliomas: a pilot study. J Cancer Res Clin Oncol.

[17] Beppu T, Kamada K, Nakamura R, Oikawa H, Takeda M, Fukuda T (2003). A phase II study of radiotherapy after hyperbaric oxygenation combined with interferon-beta and nimustine hydrochloride to treat supratentorial malignant gliomas. J Neurooncol.

[18] Ogawa K, Yoshii Y, Inoue O, Toita T, Saito A, Kakinohana Y (2003). Prospective trial of radiotherapy after hyperbaric oxygenation with chemotherapy for high-grade gliomas. Radiother Oncol.

[19] Ogawa K, Yoshii Y, Inoue O (2006). Phase II trial of radiotherapy after hyperbaric oxygenation with chemotherapy for high-grade gliomas. Br J Cancer.

[20] Yahara K, Ohguri T, Udono H, Yamamoto J, Tomura K, Onoda T (2017). Radiotherapy using IMRT boosts after hyperbaric oxygen therapy with chemotherapy for glioblastoma. J Radiat Res.

[21] Kohshi K, Yamamoto H, Nakahara A, Katoh T, Takagi M (2007). Fractionated stereotactic radiotherapy using gamma unit after hyperbaric oxygenation on recurrent high-grade gliomas. J Neurooncol.

[22] Huang L, Boling W, Zhang JH (2018). Hyperbaric oxygen therapy as adjunctive strategy in treatment of glioblastoma multiforme. Med Gas Res.

[23] Simon JM, Noël G, Chiras J (2003). Radiotherapy and chemotherapy with or without carbogen and nicotinamide in inoperable biopsy-proven glioblastoma multiforme. Radiother Oncol.

[24] van der Maazen RW, Thijssen HO, Kaanders JH (1995). Conventional radiotherapy combined with carbogen breathing and nicotinamide for malignant gliomas. Radiother Oncol.

[25] Pickles T, Graham P, Syndikus I, Rheaume DE, Duncan GG, Green A (1996). Tolerance of nicotinamide and carbogen with radiation therapy for glioblastoma. Radiother Oncol.

[26] Fatigante L, Ducci F, Cartei F, Colosimo S, Marini C, Prediletto R (1997). Carbogen and nicotinamide combined with unconventional radiotherapy in glioblastoma multiforme: a new modality treatment. Int J Radiat Oncol Biol Phys.

[27] Lambin P, Poortmans P, Menten J, Hamers HP (1997). Accelerated radiotherapy with carbogen and nicotinamide (ARCON) in high grade malignant gliomas. Radiother Oncol.

[28] Miralbell R, Mornex F, Greiner R (1999). Accelerated radiotherapy, carbogen, and nicotinamide in glioblastoma multiforme: report of European organization for research and treatment of cancer trial 22933. J Clin Oncol.

[29] Hulshof MC, Rehmann CJ, Booij J, van Royen EA, Bosch DA, González González D (1998). Lack of perfusion enhancement after administration of nicotinamide and carbogen in patients with glioblastoma: a 99mTc-HMPAO SPECT study. Radiother Oncol.

[30] Delmas C, Heliez C, Cohen-Jonathan E (2002). Farnesyltransferase inhibitor, R115777, reverses the resistance of human glioma cell lines to ionizing radiation. Int J Cancer.

[31] Moyal EC, Laprie A, Delannes M (2007). Phase I trial of tipifarnib (R115777) concurrent with radiotherapy in patients with glioblastoma multiforme. Int J Radiat Oncol Biol Phys.

[32] Cloughesy TF, Wen PY, Robins HI (2006). Phase II trial of tipifarnib in patients with recurrent malignant glioma either receiving or not receiving enzyme-inducing antiepileptic drugs: a north American brain tumor consortium study. J Clin Oncol.

[33] Lustig R, Mikkelsen T, Lesser G (2008). Phase II preradiation R115777 (tipifarnib) in newly diagnosed GBM with residual enhancing disease. Neuro Oncol.

[34] Nghiemphu PL, Wen PY, Lamborn KR (2011). A phase I trial of tipifarnib with radiation therapy, with and without temozolomide, for patients with newly diagnosed glioblastoma. Int J Radiat Oncol Biol Phys.

[35] Ducassou A, Uro-Coste E, Verrelle P (2013). αvβ3 integrin and fibroblast growth factor receptor 1 (FGFR1): prognostic factors in a phase I–II clinical trial associating continuous administration of Tipifarnib with radiotherapy for patients with newly diagnosed glioblastoma. Eur J Cancer.

[36] Nghiemphu PL, Ebiana VA, Wen P (2018). Phase I study of sorafenib and tipifarnib for recurrent glioblastoma: NABTC 05-02. J Neurooncol.

[37] Kleinberg L, Grossman SA, Piantadosi S (1999). Phase I trial to determine the safety, pharmacodynamics, and pharmacokinetics of RSR13, a novel radioenhancer, in newly diagnosed glioblastoma multiforme. J Clin Oncol.

[38] Kleinberg L, Grossman SA, Carson K (2002). Survival of patients with newly diagnosed glioblastoma multiforme treated with RSR13 and radiotherapy: results of a phase II new approaches to brain tumor therapy CNS consortium safety and efficacy study. J Clin Oncol.

[39] Del Rowe J, Scott C, Werner-Wasik M, Bahary JP, Curran WJ, Urtasun RC (2000). Single-arm, open-label phase II study of intravenously administered tirapazamine and radiation therapy for glioblastoma multiforme. J Clin Oncol.

[40] Zimbrick JD, Ward JF, Myers LS (1969). Studies on the chemical basis of cellular radiosensitization by 5-bromouracil substitution in DNA. II. Pulse- and steadystate radiolysis of bromouracil-substituted and unsubstituted DNA. Int J Radiat Biol Relat Stud Phys Chem Med.

[41] Kinsella TJ, Dobson PP, Mitchell JB, Fornace AJ (1987). Enhancement of X ray induced DNA damage by pre-treatment with halogenated pyrimidine analogs. Int J Radiat Oncol Biol Phys.

[42] Jackson D, Kinsella T, Rowland J (1987). Halogenated pyrimidines as radiosensitizers in the treatment of glioblastoma multiforme. Am J Clin Oncol.

[43] Matsutani M, Kohno T, Nagashima T (1988). Clinical trial of intravenous infusion of bromodeoxyuridine (BUdR) for radiosensitization of malignant brain tumors. Radiat Med.

[44] Greenberg HS, Chandler WF, Diaz RF (1988). Intra-arterial bromodeoxyuridine radiosensitization and radiation in treatment of malignant astrocytomas. J Neurosurg.

[45] Hegarty TJ, Thornton AF, Diaz RF (1990). Intra-arterial bromodeoxyuridine radiosensitization of malignant gliomas. Int J Radiat Oncol Biol Phys.

[46] Greenberg HS, Chandler WF, Ensminger WD (1994). Radiosensitization with carotid intra-arterial bromodeoxyuridine +/− 5-fluorouracil biomodulation for malignant gliomas. Neurology.

[47] Urtasun RC, Cosmatos D, DelRowe J (1993). Iododeoxyuridine (IUdR) combined with radiation in the treatment of malignant glioma: a comparison of short versus long intravenous dose schedules (RTOG 86-12). Int J Radiat Oncol Biol Phys.

[48] Goffman TE, Dachowski LJ, Bobo H (1992). Long-term follow-up on national cancer institute phase I/II study of glioblastoma multiforme treated with iododeoxyuridine and hyperfractionated irradiation. J Clin Oncol.

[49] Groves MD, Maor MH, Meyers C (1999). A phase II trial of high-dose bromodeoxyuridine with accelerated fractionation radiotherapy followed by procarbazine, lomustine, and vincristine for glioblastoma multiforme. Int J Radiat Oncol Biol Phys.

[50] Vokes EE, Dolan ME, Krishnasamy S (1993). 5-Fluorouracil, hydroxyurea and escalating doses of iododeoxyuridine with concomitant radiotherapy for malignant gliomas: a clinical and pharmacologic analysis. Ann Oncol.

[51] Phillips TL, Levin VA, Ahn DK (1991). Evaluation of bromodeoxyuridine in glioblastoma multiforme: a northern California cancer center phase II study. Int J Radiat Oncol Biol Phys.

[52] Prados MD, Seiferheld W, Sandler HM (2004). Phase III randomized study of radiotherapy plus procarbazine, lomustine, and vincristine with or without BUdR for treatment of anaplastic astrocytoma: final report of RTOG 9404. Int J Radiat Oncol Biol Phys.

[53] Mapelli R, Julita C, Bianchi SP, Gallina N, Lucchini R, Midulla M, Puci F, Saddi J, Trivellato S, Panizza D, De Ponti E, Arcangeli S (2021). Association between treatment-related lymphopenia and survival in glioblastoma patients following postoperative chemoradiotherapy. Strahlenther Onkol.

[54] Su JM, Thompson P, Adesina A (2014). A phase I trial of veliparib (ABT-888) and temozolomide in children with recurrent CNS tumors: a pediatric brain tumor consortium report. Neuro Oncol.

[55] Lesueur P, Chevalier F, El-Habr EA (2018). Radiosensitization effect of talazoparib, a parp inhibitor, on glioblastoma stem cells exposed to low and high linear energy transfer radiation. Sci Rep.

[56] Galia A, Calogero AE, Condorelli R (2012). PARP-1 protein expression in glioblastoma multiforme. Eur J Histochem.

[57] Baxter PA, Su JM, Onar-Thomas A (2020). A phase I/II study of veliparib (ABT-888) with radiation and temozolomide in newly diagnosed diffuse pontine glioma: a pediatric brain tumor consortium study. Neuro Oncol.

[58] Robins HI, Zhang P, Gilbert MR (2016). A randomized phase I/II study of ABT-888 in combination with temozolomide in recurrent temozolomide resistant glioblastoma: an NRG oncology RTOG group study. J Neurooncol.

[59] Hanna C, Kurian KM, Williams K (2020). Pharmacokinetics, safety, and tolerability of olaparib and temozolomide for recurrent glioblastoma: results of the phase I OPARATIC trial. Neuro Oncol.

[60] Sim HW, McDonald KL, Lwin Z (2021). A randomized phase II trial of veliparib, radiotherapy, and temozolomide in patients with unmethylated MGMT glioblastoma: the VERTU study. Neuro Oncol.

[61] Lesueur P, Lequesne J, Grellard JM (2019). Phase I/IIa study of concomitant radiotherapy with olaparib and temozolomide in unresectable or partially resectable glioblastoma: OLA-TMZ-RTE-01 trial protocol. BMC Cancer.

[62] Amato RJ, Jac J, Hernandez-McClain J (2008). Motexafin gadolinium for the treatment of metastatic renal cell carcinoma: phase II study results. Clin Genitourin Cancer.

[63] Edelman MJ, Otterson G, Leach J (2011). Multicenter phase II trial of motexafin gadolinium and pemetrexed for second-line treatment in patients with non-small cell lung cancer. J Thorac Oncol.

[64] Evens AM, Spies WG, Helenowski IB (2009). The novel expanded porphyrin, motexafin gadolinium, combined with [90Y]ibritumomab tiuxetan for relapsed/refractory non-Hodgkin’s lymphoma: preclinical findings and results of a phase I trial. Clin Cancer Res.

[65] Bradley KA, Pollack IF, Reid JM (2008). Motexafin gadolinium and involved field radiation therapy for intrinsic pontine glioma of childhood: a children’s oncology group phase I study. Neuro Oncol.

[66] Carde P, Timmerman R, Mehta MP (2001). Multicenter phase Ib/II trial of the radiation enhancer motexafin gadolinium in patients with brain metastases. J Clin Oncol.

[67] Ford JM, Seiferheld W, Alger JR (2007). Results of the phase I dose-escalating study of motexafin gadolinium with standard radiotherapy in patients with glioblastoma multiforme. Int J Radiat Oncol Biol Phys.

[68] Hashemy SI, Ungerstedt JS, Zahedi Avval F, Holmgren A (2006). Motexafin gadolinium, a tumor-selective drug targeting thioredoxin reductase and ribonucleotide reductase. J Biol Chem.

[69] Wu GN, Ford JM, Alger JR (2006). MRI measurement of the uptake and retention of motexafin gadolinium in glioblastoma multiforme and uninvolved normal human brain. J Neurooncol.

[70] Brachman DG, Pugh SL, Ashby LS (2015). Phase 1/2 trials of temozolomide, motexafin gadolinium, and 60-Gy fractionated radiation for newly diagnosed supratentorial glioblastoma multiforme: final results of RTOG 0513. Int J Radiat Oncol Biol Phys.

[71] Metcalf BW, Bey P, Danzin C, Jung MJ, Casara P, Vevert JP (1978). Catalytic irreversible inhibition of mammalian ornithine decarboxylase by substrate and product analogues. J Am Chem Soc.

[72] Arundel CM, Nishioka K, Tofilon PJ (1988). Effects of alpha-difluoromethylornithine-induced polyamine depletion on the radiosensitivity of a human colon carcinoma cell line. Radiat Res.

[73] Prados MD, Wara WM, Sneed PK (2001). Phase III trial of accelerated hyperfractionation with or without difluromethylornithine (DFMO) versus standard fractionated radiotherapy with or without DFMO for newly diagnosed patients with glioblastoma multiforme. Int J Radiat Oncol Biol Phys.

[74] Wildfant I, Grundel O, Schmoll H-J (1995). 582 The role of beta-interferon as a radiosensitizer in therapy-refractory metastases from solid tumors—a phase-II-study. Eur J Cancer.

[75] Dillman RO, Wiemann M, Oldham RK (1995). Interferon alpha-2a and external beam radiotherapy in the initial management of patients with glioma: a pilot study of the national biotherapy study group. Cancer Biother.

[76] Lippman SM, Kavanagh JJ, Paredes-Espinoza M (1993). 13-cis-retinoic acid plus interferon-alpha 2a in locally advanced squamous cell carcinoma of the cervix. J Natl Cancer Inst.

[77] Dillman RO, Shea WM, Tai DF (2001). Interferon-alpha2a and 13-cis-retinoic acid with radiation treatment for high-grade glioma. Neuro Oncol.

[78] Sanli T, Liu C, Rashid A (2011). Lovastatin sensitizes lung cancer cells to ionizing radiation: modulation of molecular pathways of radioresistance and tumor suppression. J Thorac Oncol.

[79] Nübel T, Damrot J, Roos WP, Kaina B, Fritz G (2006). Lovastatin protects human endothelial cells from killing by ionizing radiation without impairing induction and repair of DNA double-strand breaks. Clin Cancer Res.

[80] Larner J, Jane J, Laws E, Packer R, Myers C, Shaffrey M (1998). A phase I–II trial of lovastatin for anaplastic astrocytoma and glioblastoma multiforme. Am J Clin Oncol.

[81] Happold C, Gorlia T, Nabors LB (2018). Do statins, ACE inhibitors or sartans improve outcome in primary glioblastoma?. J Neurooncol.

[82] Haritz D, Gabel D, Huiskamp R (1994). Clinical phase-I study of Na2B12H11SH (BSH) in patients with malignant glioma as precondition for boron neutron capture therapy (BNCT). Int J Radiat Oncol Biol Phys.

[83] Palmer MR, Goorley JT, Kiger WS (2002). Treatment planning and dosimetry for the Harvard-MIT phase I clinical trial of cranial neutron capture therapy. Int J Radiat Oncol Biol Phys.

[84] Coderre JA, Elowitz EH, Chadha M (1997). Boron neutron capture therapy for glioblastoma multiforme using p-boronophenylalanine and epithermal neutrons: trial design and early clinical results. J Neurooncol.

[85] Takagaki M, Oda Y, Miyatake S (1997). Boron neutron capture therapy: preliminary study of BNCT with sodium borocaptate (Na2B1 2H1 1SH) on glioblastoma. J Neurooncol.

[86] Chadha M, Capala J, Coderre JA (1998). Boron neutron-capture therapy (BNCT) for glioblastoma multiforme (GBM) using the epithermal neutron beam at the Brookhaven national laboratory. Int J Radiat Oncol Biol Phys.

[87] Capala J, Stenstam BH, Sköld K (2003). Boron neutron capture therapy for glioblastoma multiforme: clinical studies in Sweden. J Neurooncol.

[88] Kageji T, Nagahiro S, Mizobuchi Y, Toi H, Nakagawa Y, Kumada H (2004). Radiation injury of boron neutron capture therapy using mixed epithermal- and thermal neutron beams in patients with malignant glioma. Appl Radiat Isot.

[89] Kiger WS, Lu XQ, Harling OK (2004). Preliminary treatment planning and dosimetry for a clinical trial of neutron capture therapy using a fission converter epithermal neutron beam. Appl Radiat Isot.

[90] Yamamoto T, Matsumura A, Nakai K (2004). Current clinical results of the Tsukuba BNCT trial. Appl Radiat Isot.

[91] Miyatake S, Kawabata S, Kajimoto Y (2005). Modified boron neutron capture therapy for malignant gliomas performed using epithermal neutron and two boron compounds with different accumulation mechanisms: an efficacy study based on findings on neuroimages. J Neurosurg.

[92] Miyatake S, Kawabata S, Yokoyama K (2009). Survival benefit of Boron neutron capture therapy for recurrent malignant gliomas. J Neurooncol.

[93] Kawabata S, Miyatake S, Kuroiwa T (2009). Boron neutron capture therapy for newly diagnosed glioblastoma. J Radiat Res.

[94] Kawabata S, Miyatake S, Hiramatsu R (2011). Phase II clinical study of boron neutron capture therapy combined with X-ray radiotherapy/temozolomide in patients with newly diagnosed glioblastoma multiforme-study design and current status report. Appl Radiat Isot.

[95] Aiyama H, Nakai K, Yamamoto T (2011). A clinical trial protocol for second line treatment of malignant brain tumors with BNCT at University of Tsukuba. Appl Radiat Isot.

[96] Diaz AZ (2003). Assessment of the results from the phase I/II boron neutron capture therapy trials at the Brookhaven national laboratory from a clinician’s point of view. J Neurooncol.

[97] Kankaanranta L, Seppälä T, Koivunoro H (2011). L-boronophenylalanine-mediated boron neutron capture therapy for malignant glioma progressing after external beam radiation therapy: a phase I study. Int J Radiat Oncol Biol Phys.

[98] H-Stenstam B, Pellettieri L, Sköld K, Rezaei A, Brun A (2007). Neuropathological postmortem evaluation of BNCT for GBM. Acta Neurol Scand.

[99] Kageji T, Mizobuchi Y, Nagahiro S, Nakagawa Y, Kumada H (2014). Correlation between radiation dose and histopathological findings in patients with gliblastoma treated with boron neutron capture therapy (BNCT). Appl Radiat Isot.

[100] Henriksson R, Capala J, Michanek A (2008). Boron neutron capture therapy (BNCT) for glioblastoma multiforme: a phase II study evaluating a prolonged high-dose of boronophenylalanine (BPA). Radiother Oncol.

[101] Sköld K, H-Stenstam B, Diaz AZ, Giusti V, Pellettieri L, Hopewell JW (2010). Boron neutron capture therapy for glioblastoma multiforme: advantage of prolonged infusion of BPA-f. Acta Neurol Scand.

[102] Hopewell JW, Gorlia T, Pellettieri L, Giusti V, H-Stenstam B, Sköld K (2011). Boron neutron capture therapy for newly diagnosed glioblastoma multiforme: an assessment of clinical potential. Appl Radiat Isot.

[103] Sander A, Wosniok W, Gabel D (2014). Case numbers for a randomized clinical trial of boron neutron capture therapy for glioblastoma multiforme. Appl Radiat Isot.

[104] Lautenschlaeger FS, Dumke R, Schymalla M (2021). Comparison of carbon ion and photon reirradiation for recurrent glioblastoma. Strahlenther Onkol.

[105] Stummer W, Pichlmeier U, Meinel T, Wiestler OD, Zanella F, Reulen HJ (2006). Fluorescence-guided surgery with 5-aminolevulinic acid for resection of malignant glioma: a randomised controlled multicentre phase III trial. Lancet Oncol.

[106] Beck TJ, Kreth FW, Beyer W (2007). Interstitial photodynamic therapy of nonresectable malignant glioma recurrences using 5-aminolevulinic acid induced protoporphyrin IX. Lasers Surg Med.

[107] Mahmoudi K, Garvey KL, Bouras A (2019). 5-aminolevulinic acid photodynamic therapy for the treatment of high-grade gliomas. J Neurooncol.

[108] Stepp H, Stummer W (2018). 5-ALA in the management of malignant glioma. Lasers Surg Med.

[109] Ueta K, Yamamoto J, Tanaka T, Nakano Y, Kitagawa T, Nishizawa S (2017). 5-Aminolevulinic acid enhances mitochondrial stress upon ionizing irradiation exposure and increases delayed production of reactive oxygen species and cell death in glioma cells. Int J Mol Med.

[110] Yamamoto J, Ogura SI, Shimajiri S (2015). 5-Aminolevulinic acid-induced protoporphyrin IX with multi-doseionizing irradiation enhances host antitumor response and strongly inhibits tumor growth in experimental glioma in vivo. Mol Med Rep.

[111] Kitagawa T, Yamamoto J, Tanaka T, Nakano Y, Akiba D, Ueta K, Nishizawa S (2015). 5-Aminolevulinic acid strongly enhances delayed intracellular production of reactive oxygen species (ROS) generated by ionizing irradiation: quantitative analyses and visualization of intracellular ROS production in glioma cells in vitro. Oncol Rep.

[112] Wang B, Cvetkovic D, Gupta R, Chen L, Ma CMC, Zhang Q, Zeng J (2015). Radiation therapy combined with 5-aminolevulinic acid: a preliminary study with an in vivo mouse model implanted with human PC-3 tumor cells. Int J Radiat Oncol Biol Phys.

[113] Panetta JV, Cvetkovic D, Chen X, Chen L, Ma CC (2020). Radiodynamic therapy using 15-MV radiation combined with 5-aminolevulinic acid and carbamide peroxide for prostate cancer in vivo. Phys Med Biol.

[114] Gaber M, Selim H, El-Nahas T (2013). Prospective study evaluating the radiosensitizing effect of reduced doses of temozolomide in the treatment of Egyptian patients with glioblastoma multiforme. Cancer Manag Res.

[115] Hegi ME, Diserens AC, Gorlia T (2005). MGMT gene silencing and benefit from temozolomide in glioblastoma. N Engl J Med.

[116] Kim N, Chang JS, Wee CW, Kim IA, Chang JH, Lee HS (2020). Validation and optimization of a web-based nomogram for predicting survival of patients with newly diagnosed glioblastoma. Strahlenther Onkol.

[117] Minea RO, Duc TC, Swenson SD (2020). Developing a clinically relevant radiosensitizer for temozolomide-resistant gliomas. PLoS ONE.

[118] Weller M, Le Rhun E (2020). How did lomustine become standard of care in recurrent glioblastoma?. Cancer Treat Rev.

[119] Miller AC, Blakely WF (1992). Inhibition of glutathione reductase activity by a carbamoylating nitrosourea: effect on cellular radiosensitivity. Free Radic Biol Med.

[120] Sinclair WK, Morton RA (1966). X-ray sensitivity during the cell generation cycle of cultured Chinese Hamster cells. Radiat Res.

[121] Murphy C, Pickles T, Knowling M, Thiesse B (2002). Concurrent modified PCV chemotherapy and radiotherapy in newly diagnosed grade IV astrocytoma. J Neurooncol.

[122] Schmidt F, Fischer J, Herrlinger U, Dietz K, Dichgans J, Weller M (2006). PCV chemotherapy for recurrent glioblastoma. Neurology.

[123] Weller M, van den Bent M, Preusser M (2021). EANO guidelines on the diagnosis and treatment of diffuse gliomas of adulthood. Nat Rev Clin Oncol.

[124] Dahl WN, Oftebro R, Pettersen EO, Brustad T (1976). Inhibitory and cytotoxic effects of oncovin (vincristine sulfate) on cells of human line NHIK 3025. Cancer Res.

[125] Roberts PB (1979). Radiosensitization of E. coli B/r by the cytotoxic agent procarbazine: a hypoxic cell sensitizer preferentially toxic to aerobic cells and easily oxidized. Br J Cancer.

[126] Buckner JC, Shaw EG, Pugh SL (2016). Radiation plus procarbazine, CCNU, and vincristine in low-grade glioma. N Engl J Med.

[127] Cairncross G, Wang M, Shaw E (2013). Phase III trial of chemoradiotherapy for anaplastic oligodendroglioma: long-term results of RTOG 9402. J Clin Oncol.

[128] Kim SH, Yoo H, Chang JH (2018). Procarbazine and CCNU chemotherapy for recurrent glioblastoma with MGMT promoter methylation. J Korean Med Sci.

[129] Liebmann J, Cook JA, Fisher J, Teague D, Mitchell JB (1994). In vitro studies of taxol as a radiation sensitizer in human tumor cells. J Natl Cancer Inst.

[130] Glantz MJ, Choy H, Kearns CM, Akerley W, Egorin MJ (1995). Weekly, outpatient paclitaxel and concurrent cranial irradiation in adults with brain tumors: preliminary results and promising directions. Semin. Oncol..

[131] Glantz MJ, Choy H, Kearns CM (1996). Phase I study of weekly outpatient paclitaxel and concurrent cranial irradiation in adults with astrocytomas. J Clin Oncol.

[132] Fetell MR, Grossman SA, Fisher JD (1997). Preirradiation paclitaxel in glioblastoma multiforme: efficacy, pharmacology, and drug interactions. New approaches to brain tumor therapy central nervous system consortium. J Clin Oncol.

[133] Fountzilas G, Karavelis A, Capizzello A (1999). Radiation and concomitant weekly administration of paclitaxel in patients with glioblastoma multiforme. A phase II study. J Neurooncol.

[134] Lederman G, Wronski M, Arbit E (2000). Treatment of recurrent glioblastoma multiforme using fractionated stereotactic radiosurgery and concurrent paclitaxel. Am J Clin Oncol.

[135] Ashamalla H, Zaki B, Mokhtar B (2007). Fractionated stereotactic radiotherapy boost and weekly paclitaxel in malignant gliomas clinical and pharmacokinetics results. Technol Cancer Res Treat.

[136] Li C, Ke S, Wu QP (2000). Tumor irradiation enhances the tumor-specific distribution of poly(L-glutamic acid)-conjugated paclitaxel and its antitumor efficacy. Clin Cancer Res.

[137] Jeyapalan S, Boxerman J, Donahue J (2014). Paclitaxel poliglumex, temozolomide, and radiation for newly diagnosed high-grade glioma: a Brown university oncology group study. Am J Clin Oncol.

[138] Elinzano H, Glantz M, Mrugala M (2018). PPX and concurrent radiation for newly diagnosed glioblastoma without MGMT methylation: a randomized phase II study: brUOG 244. Am J Clin Oncol.

[139] Ojima E, Inoue Y, Watanabe H (2006). The optimal schedule for 5-fluorouracil radiosensitization in colon cancer cell lines. Oncol Rep.

[140] Valdes G, Iwamoto KS (2013). Re-evaluation of cellular radiosensitization by 5-fluorouracil: high-dose, pulsed administration is effective and preferable to conventional low-dose, chronic administration. Int J Radiat Biol.

[141] Shapiro WR, Green SB, Burger PC (1992). A randomized comparison of intra-arterial versus intravenous BCNU, with or without intravenous 5-fluorouracil, for newly diagnosed patients with malignant glioma. J Neurosurg.

[142] Grunda JM, Fiveash J, Palmer CA (2010). Rationally designed pharmacogenomic treatment using concurrent capecitabine and radiotherapy for glioblastoma; gene expression profiles associated with outcome. Clin Cancer Res.

[143] Larner JM, Phillips CD, Dion JE, Jensen ME, Newman SA, Jane JA (1995). A phase 1–2 trial of superselective carboplatin, low-dose infusional 5-fluorouracil and concurrent radiation for high-grade gliomas. Am J Clin Oncol.

[144] Menei P, Venier MC, Gamelin E (1999). Local and sustained delivery of 5-fluorouracil from biodegradable microspheres for the radiosensitization of glioblastoma: a pilot study. Cancer.

[145] Menei P, Benoit JP (2003). Implantable drug-releasing biodegradable microspheres for local treatment of brain glioma. Acta Neurochir Suppl.

[146] Sigmond J, Honeywell RJ, Postma TJ (2009). Gemcitabine uptake in glioblastoma multiforme: potential as a radiosensitizer. Ann Oncol.

[147] Pauwels B, Korst AE, Lardon F, Vermorken JB (2005). Combined modality therapy of gemcitabine and radiation. Oncologist.

[148] Metro G, Fabi A, Mirri MA (2010). Phase II study of fixed dose rate gemcitabine as radiosensitizer for newly diagnosed glioblastoma multiforme. Cancer Chemother Pharmacol.

[149] Fabi A, Mirri A, Felici A (2008). Fixed dose-rate gemcitabine as radiosensitizer for newly diagnosed glioblastoma: a dose-finding study. J Neurooncol.

[150] Wick W, Hermisson M, Kortmann RD (2002). Neoadjuvant gemcitabine/treosulfan chemotherapy for newly diagnosed glioblastoma: a phase II study. J Neurooncol.

[151] Weller M, Streffer J, Wick W (2001). Preirradiation gemcitabine chemotherapy for newly diagnosed glioblastoma. A phase II study. Cancer.

[152] Gertler SZ, MacDonald D, Goodyear M (2000). NCIC-CTG phase II study of gemcitabine in patients with malignant glioma (IND.94). Ann Oncol.

[153] Jiang Z, Pflug K, Usama SM (2019). Cyanine-gemcitabine conjugates as targeted theranostic agents for glioblastoma tumor cells. J Med Chem.

[154] Bastiancich C, Lemaire L, Bianco J (2018). Evaluation of lauroyl-gemcitabine-loaded hydrogel efficacy in glioblastoma rat models. Nanomedicine (Lond).

[155] Boeckman HJ, Trego KS, Turchi JJ (2005). Cisplatin sensitizes cancer cells to ionizing radiation via inhibition of nonhomologous end joining. Mol Cancer Res.

[156] Buckner JC, Ballman KV, Michalak JC (2006). Phase III trial of carmustine and cisplatin compared with carmustine alone and standard radiation therapy or accelerated radiation therapy in patients with glioblastoma multiforme: north central cancer treatment group 93-72-52 and southwest oncology group 9503 trials. J Clin Oncol.

[157] van den Bent MJ, Pronk L, Sillevis Smitt PA, Vecht CJ, Eskens FA, Verweij J (1999). Phase II study of weekly dose-intensified cisplatin chemotherapy with oral etoposide in recurrent glioma. J Neurooncol.

[158] Enríquez Pérez J, Fritzell S, Kopecky J, Visse E, Darabi A, Siesjö P (2019). The effect of locally delivered cisplatin is dependent on an intact immune function in an experimental glioma model. Sci Rep.

[159] Chen JLY, Pan CK, Lin YL (2021). Preclinical evaluation of PEGylated liposomal doxorubicin as an effective radiosensitizer in chemoradiotherapy for lung cancer. Strahlenther Onkol.

[160] Charest G, Sanche L, Fortin D, Mathieu D, Paquette B (2013). Optimization of the route of platinum drugs administration to optimize the concomitant treatment with radiotherapy for glioblastoma implanted in the Fischer rat brain. J Neurooncol.

[161] Charest G, Sanche L, Fortin D, Mathieu D, Paquette B (2012). Glioblastoma treatment: bypassing the toxicity of platinum compounds by using liposomal formulation and increasing treatment efficiency with concomitant radiotherapy. Int J Radiat Oncol Biol Phys.

[162] Elleaume H, Barth RF, Rousseau J (2020). Radiation therapy combined with intracerebral convection-enhanced delivery of cisplatin or carboplatin for treatment of the F98 rat glioma. J Neurooncol.

[163] Niyazi M, Harter PN, Hattingen E (2016). Bevacizumab and radiotherapy for the treatment of glioblastoma: brothers in arms or unholy alliance?. Oncotarget.

[164] Das S, Marsden PA (2013). Angiogenesis in glioblastoma. N Engl J Med.

[165] McGee MC, Hamner JB, Williams RF (2010). Improved intratumoral oxygenation through vascular normalization increases glioma sensitivity to ionizing radiation. Int J Radiat Oncol Biol Phys.

[166] Schernberg A, Dhermain F, Ammari S (2018). Reirradiation with concurrent bevacizumab for recurrent high-grade gliomas in adult patients. Cancer Radiother.

[167] Vredenburgh JJ, Desjardins A, Herndon JE (2007). Bevacizumab plus irinotecan in recurrent glioblastoma multiforme. J Clin Oncol.

[168] Chinot OL, Wick W, Mason W (2014). Bevacizumab plus radiotherapy-temozolomide for newly diagnosed glioblastoma. N Engl J Med.

[169] Gilbert MR, Dignam JJ, Armstrong TS (2014). A randomized trial of bevacizumab for newly diagnosed glioblastoma. N Engl J Med.

[170] Wick W, Gorlia T, Bendszus M (2017). Lomustine and bevacizumab in progressive glioblastoma. N Engl J Med.

[171] Wick W (2021). Gliome, S2k-Leitlinie.

[172] Floeth FW, Sabel M, Ewelt C, Stummer W, Felsberg J, Reifenberger G, Steiger HJ, Stoffels G, Coenen HH, Langen KJ (2011). Comparison of (18)F-FET PET and 5-ALA fluorescence in cerebral gliomas. Eur J Nucl Med Mol Imaging.

[173] Picart T, Berhouma M, Dumot C, Pallud J, Metellus P, Armoiry X, Guyotat J (2019). Optimization of high-grade glioma resection using 5-ALA fluorescence-guided surgery: a literature review and practical recommendations from the neuro-oncology club of the French society of neurosurgery. Neurochirurgie.

[174] Johansson A, Palte G, Schnell O, Tonn JC, Herms J, Stepp H (2010). 5-Aminolevulinic acid-induced protoporphyrin IX levels in tissue of human malignant brain tumors. Photochem Photobiol.

[175] Zhang X, Bobeica M, Unger M (2021). Focused ultrasound radiosensitizes human cancer cells by enhancement of DNA damage. Strahlenther Onkol.

